# Polymyositis and Severe Rhabdomyolysis in the Context of Influenza and COVID-19 Vaccination: A Case Report

**DOI:** 10.7759/cureus.66957

**Published:** 2024-08-15

**Authors:** Angela Ghiletchi, Rodrigo Leão, Carolina Coelho, Inês Ferreira, Rita Bernardino

**Affiliations:** 1 Internal Medicine, Centro Hospitalar de Lisboa Central, Lisbon, PRT; 2 Internal Medicine, NOVA Medical School, Lisbon, PRT

**Keywords:** vaccine-induced rhabdomyolysis, severe rhabdomyolysis, polymyositis, acute kidney injury, influenza vaccine, covid-19 vaccine

## Abstract

Rhabdomyolysis is characterized by muscle breakdown and the release of muscle enzymes into the bloodstream, which can lead to acute kidney injury (AKI) and electrolyte imbalances. This case report details a 52-year-old male who developed severe rhabdomyolysis and polymyositis following influenza and SARS-COV-2 vaccinations. Presenting with severe muscle pain and elevated creatine kinase (CK) levels, the patient’s condition was managed with aggressive hydration and supportive care, resulting in significant recovery. While vaccine-related adverse effects such as myositis and rhabdomyolysis are rare, this case underscores the need for vigilance in monitoring post-vaccination complications and highlights the importance of recognizing and promptly treating vaccine-associated inflammatory myopathies to prevent severe complications. The findings contribute to the growing body of literature on vaccine-induced myopathies and emphasize the necessity of a multidisciplinary approach in managing such complex cases.

## Introduction

Rhabdomyolysis is a condition characterized by the breakdown of muscle tissue leading to the release of muscle contents such as myoglobin and creatine kinase (CK) into the bloodstream, which can result in severe complications, including acute kidney injury (AKI) and electrolyte imbalances [1]. While the etiology of rhabdomyolysis is multifactorial, recent literature has noted associations with viral infections and vaccinations [2,3]. Polymyositis, an inflammatory myopathy, can precipitate rhabdomyolysis and presents with muscle weakness and elevated serum muscle enzymes [4].

The introduction of COVID-19 vaccines has brought to light various adverse effects, with reports of myositis and rhabdomyolysis as soon as the day after the immunization [5,6]. Although vaccines like Pfizer-BioNTech (BNT162b2) are generally safe, rare instances of vaccine-induced inflammatory myopathies and rhabdomyolysis have been documented [7,8]. The relationship between immunization and autoimmune phenomena, while uncommon, requires awareness and prompt management to mitigate complications.

## Case presentation

A 52-year-old Caucasian male was brought to the emergency department (ED) after being found immobile on the floor for more than 10 hours. The patient experienced sudden severe pain and muscle weakness in his lower extremities while standing, leading to a fall. He crawled himself a few meters but remained on the floor due to pain and decreased strength. He denied loss of consciousness, vertigo, or dizziness, and there was no involuntary loss of sphincter control or recent trauma.

His medical history included compensated schizoaffective disorder, non-insulin-dependent diabetes mellitus, hypertension, asthma, and dyslipidemia. He was on several medications including metformin, montelukast, empagliflozin, gliclazide, low-dose rosuvastatin, propranolol, clozapine, biperiden, long-acting haloperidol, and a combined corticosteroid and beta-2 agonist inhaler. There were no recent medication changes in the past year, but he had received influenza (annually since 2021) and SARS-COV-2 (Pfizer BionTech, fifth immunization) vaccines two days prior.

On arrival, the patient showed repetitive speech and fleeting attention but no other neurological signs. His vital signs included a blood pressure of 94/60 mmHg, a heart rate of 72 bpm in sinus rhythm, and signs of dehydration. Physical examination of the lower extremities revealed no abnormalities besides diminished muscle strength with inability to walk, and although complaining of light myalgia, he denied pain upon palpation.

Initial blood work (Table 1) showed mild leukocytosis with neutrophilia, AKI, elevated liver enzymes and L-lactate dehydrogenase, hypernatremia, hyperphosphatemia, hypermagnesemia, elevated C-reactive protein, slightly elevated troponin, and severe rhabdomyolysis with CK levels over 400,000 U/L. Urinalysis showed dark brown urine, a pH of six, proteinuria, ketonuria, and glycosuria. A head computed tomography (CT) scan was also performed and revealed no abnormal signs.

**Table 1:** Altered laboratory parameters evaluated upon admission to the emergency department

Laboratory parameter	Value	Normal range
Leukocytes	14.05 x 10^9^/L	4.5-11.0x10^9^/L
Neutrophils	11.45 x 10^9^/L	2.0-8.5x10^9^/L
Ureia (blood)	117 mg/dl	16.6-48.5 mg/dL
Creatinine (blood)	2.59 mg/dl	0.67-1.17 mg/dL
Aspartate transaminase	1806 U/L	<40 U/L
Alanine transaminase	380 U/L	<41
L-lactate dehydrogenase	2409 U/L	135-225 U/L
Sodium (blood)	149 mEq/L	136-145 mEq/L
Phosphate	8.3 mg/dL	2.50-4.50 mg/dL
Magnesium	3.93 mg/dL	1.6-2.6 mg/dL
C-reactive protein	92 mg/L	1.6-2.6 mg/L
Troponin I (TnI)	102 ng/L	<14
Creatine kinase	>400.000 U/L	<190 U/L
Erythrocyte sedimentation rate	53 mm/H	<20

The patient was admitted for intravenous hydration and further investigation. An abdominal ultrasound showed no pathological findings, and blood and urine cultures were negative. Tests for hepatotropic viruses, including HIV, HBV, and HCV, were negative (Table 2). Liver enzymes decreased during treatment and the liver-specific autoimmune panel (Table 3) was also negative, indicating that the liver injury was due to rhabdomyolysis. Thyroid function was normal.

**Table 2:** Hepatotropic virus panel HBs antigen: hepatitis B surface antigen; HCV: hepatitis C virus; HIV: human immunodeficiency virus; CMV: cytomegalovirus; HAV: hepatitis A virus; HEV: hepatitis E

Parameter	Result
HBs antigen	Negative
HCV total antibody	Negative
HIV 1+2 antibody	Negative
CMV IgG antibody	Negative
CMV IgM antibody	Negative
HAV total antibody	Negative
HAV IgM antibody	Negative
HEV polymerase chain reaction	Negative
Heterophile antibodies	Negative
Epstein-Barr viral capsid antibody, early antigen antibody, and nuclear antigen antibody	Negative

**Table 3:** Initial autoimmune panel

Parameter	Result
Anti-smooth muscle antibody	Negative
Anti-mitochondrial M2 antibody	Negative
Anti liver/kidney microsome antibody	Negative
Anti-cytosol 1 antibody	Negative
Anti-gp210 antibody	Negative
Anti-sp100 antibody	Negative
Anti-SLA/LP antibody	Negative

Despite initially elevated troponin, EKG and echocardiogram were normal, with no signs of ischemia. A lumbosacral CT scan ruled out medullary or radicular compression. The electromyogram showed no pathological findings.

During his hospital stay, the patient showed complete regression of the myalgia and no new symptoms. AKI, dehydration, and rhabdomyolysis were treated with intravenous 9% sodium chloride and oral hydration. He was discharged with normalized kidney function and almost normalized CK levels. Rosuvastatin was resumed upon discharge.

Three months post-discharge, the patient maintained normal kidney function, showed no signs of rhabdomyolysis, and had no muscle, bone, or joint pain. The polymyositis autoimmune panel, taken three months after the acute event, came back positive for PM/Scl75. Thus, after extensive investigation, it was concluded that the patient developed polymyositis and severe rhabdomyolysis due to recent influenza and SARS-COV-2 immunization and was referred to the Autoimmune Clinic to follow up on the polymyositis diagnosis, awaiting re-evaluation.

## Discussion

This case highlights the intersection of rhabdomyolysis and polymyositis in the context of recent vaccinations for influenza and SARS-COV-2. The patient’s presentation, with severe muscle pain and weakness, which led to a fall and biochemical markers indicative of rhabdomyolysis, aligns with documented cases of vaccine-induced myopathies [5,7]. The patient's elevated CK levels, AKI, and absence of external trauma or recent medication changes suggest a direct link to the recent vaccinations.

While vaccines are crucial in controlling infectious diseases, their role in triggering autoimmune responses such as myositis and rhabdomyolysis is increasingly recognized [9]. In this case, the onset of symptoms shortly after vaccination supports the hypothesis of an immunization-related adverse event. Previous studies have noted similar occurrences, where the immune response to the vaccine potentially exacerbates underlying susceptibilities, leading to muscle breakdown and systemic complications [4,10].

One potential confounding factor in this case is the patient's use of low-dose rosuvastatin, a statin known to occasionally cause rhabdomyolysis [11]. However, the patient had been on rosuvastatin for over five years without issues, and such severe CK elevation due to this statin use is improbable [2].

The management of this patient involved prompt recognition of rhabdomyolysis and aggressive hydration to prevent further renal damage. This aligns with established treatment protocols emphasizing the importance of early intervention in mitigating the progression to severe AKI [1]. The patient’s recovery, marked by normalization of kidney function and CK levels, underscores the efficacy of timely and appropriate treatment.

This case also underscores the necessity of monitoring for adverse reactions post-vaccination such as myalgias and not dismiss them simply as vague symptoms. The documented increase in inflammatory myopathies following COVID-19 vaccination [7,8] highlights the need for healthcare providers to be vigilant in identifying and managing such rare but serious events.

## Conclusions

We presented the case of a patient who suffered a severe case of rhabdomyolysis due to polymyositis and accompanying AKI. After other causes were excluded the vaccination against COVID-19 and seasonal Influenza viruses was assumed as the etiology. Although variable in incidence and severity, there are numerous reports of such links in the literature. However, unlike many others, our case presented with mild symptoms and organ injury despite the high CK values. Symptoms ameliorated and the AKI resolved within the first days of treatment, without the need for hemodialysis or other organ support systems. The scope of this case report is to raise awareness that while vaccines play an indispensable role in public health, recognizing and addressing their rare adverse effects is critical to avoid poor outcomes.

## References

[1] Cabral BM, Edding SN, Portocarrero JP, Lerma EV (2020). Rhabdomyolysis. Dis Mon.

[2] Safitri N, Alaina MF, Pitaloka DA, Abdulah R (2021). A narrative review of statin-induced rhabdomyolysis: molecular mechanism, risk factors, and management. Drug Healthc Patient Saf.

[3] Callado RB, Carneiro TG, Parahyba CC, Lima Nde A, da Silva Junior GB, Daher Ede F (2013). Rhabdomyolysis secondary to influenza A H1N1 vaccine resulting in acute kidney injury. Travel Med Infect Dis.

[4] Kim JH, Kim JH, Woo CG (2022). Clinicopathological characteristics of inflammatory myositis induced by COVID-19 vaccine (Pfizer-BioNTech BNT162b2): a case report. J Korean Med Sci.

[5] Nassar M, Chung H, Dhayaparan Y (2021). COVID-19 vaccine induced rhabdomyolysis: case report with literature review. Diabetes Metab Syndr.

[6] Dighriri IM, Alhusayni KM, Mobarki AY (2022). Pfizer-BioNTech COVID-19 vaccine (BNT162b2) side effects: a systematic review. Cureus.

[7] Syrmou V, Liaskos C, Ntavari N (2023). COVID-19 vaccine-associated myositis: a comprehensive review of the literature driven by a case report. Immunol Res.

[8] Abukhalil AD, Shatat SS, Abushehadeh RR, Al-Shami N, Naseef HA, Rabba A (2023). Side effects of Pfizer/BioNTech (BNT162b2) COVID-19 vaccine reported by the Birzeit University community. BMC Infect Dis.

[9] Conticini E, d'Alessandro M, Grazzini S (2022). Relapses of idiopathic inflammatory myopathies after vaccination against COVID-19: a real-life multicenter Italian study. Intern Emerg Med.

[10] Raman KS, Chandrasekar T, Reeve RS, Roberts ME, Kalra PA (2006). Influenza vaccine-induced rhabdomyolysis leading to acute renal transplant dysfunction. Nephrol Dial Transplant.

[11] Montastruc JL (2023). Rhabdomyolysis and statins: a pharmacovigilance comparative study between statins. Br J Clin Pharmacol.

